# Evaluation of Arsenic Concentrations in Water and Milk and Their Association with DNA Fragmentation in Lymphocytes in Goats in the Comarca Lagunera

**DOI:** 10.3390/ani16081218

**Published:** 2026-04-16

**Authors:** Ana Graciela Martínez-Delgado, Oscar Ángel-García, Viridiana Contreras-Villarreal, Guadalupe Calderón-Leyva, Javier Morán-Martínez, Nadia Denys Betancourt-Martínez, Jessica María Flores-Salas, Alan Sebastián Alvarado-Espino, Fernando Arellano-Rodríguez

**Affiliations:** 1 Departament of Animal Reproduction, Antonio Narro Agricultural Autonomous University, Laguna Unit, Av. s/n Col. Valle Verde, Torreón 27053, Coahuila, Mexico; anagracielamartinezd@gmail.com (A.G.M.-D.); alanalvaes@gmail.com (A.S.A.-E.); 2 Departament of Veterinary Medical Sciences, Antonio Narro Agricultural Autonomous University, Laguna Unit, Av. s/n Col. Valle Verde, Torreón 27053, Coahuila, Mexico; angelgarciao@hotmail.com (O.Á.-G.); dra.viridianac@gmail.com (V.C.-V.); gpecalderon06@gmail.com (G.C.-L.); jesflor13@hotmail.com (J.M.F.-S.); 3 Departament of Celullar Biology and Ultrastructure, Faculty of Medicine, Autonomous of Coahuila University, Torreón Unit, Av. Morelos 900-Oriente, Primero de Cobián Centro, Torreón 27000, Coahuila, Mexico; javiermoranmartinez@uadec.edu.mx (J.M.-M.); nabetancourtm@uadec.edu.mx (N.D.B.-M.)

**Keywords:** goats, arsenic, comet assay, DNA damage, lymphocytes, Comarca Lagunera

## Abstract

The Comarca Lagunera region faces groundwater quality issues due to elevated concentrations of heavy metals that exceed permissible limits set by Mexican regulations. This study aimed to evaluate arsenic concentrations in drinking water and goat milk, as well as their possible association with DNA fragmentation in goat lymphocytes in the Comarca Lagunera (Durango and Coahuila, Northern Mexico). Water, milk, and blood samples were collected from 120 goats (*Capra hircus*) and analyzed using atomic absorption spectrophotometry (water and milk samples) and the comet assay (blood). Arsenic concentration in drinking water varied among locations, with the highest value detected in El Venado (San Pedro, Coahuila), while other sites showed concentrations close to permissible limits. Arsenic concentrations in goat milk were generally low and mostly below the LOQ, which limited the ability to assess arsenic transfer into milk. DNA fragmentation was observed in lymphocytes; however, no statistically significant association was found between arsenic concentrations and DNA damage. These results indicate that, under the conditions of this study, DNA damage cannot be directly attributed to arsenic exposure and may be influenced by other environmental or biological factors. Further studies with larger sample sizes and additional variables are recommended.

## Simple Summary

Arsenic (As) is a toxic element commonly found in groundwater in regions such as the Comarca Lagunera, Mexico. This study evaluated arsenic levels in drinking water and goat milk, as well as their possible association with DNA damage in goat lymphocytes. Arsenic concentrations in water varied among locations, with some values exceeding recommended limits. In contrast, arsenic levels in goat milk were generally low and mostly below the limit of quantification (LOQ), limiting the assessment of arsenic transfer into milk. Although DNA damage was observed, no statistically significant association was found between arsenic levels and the extent of DNA damage. Therefore, the observed effects cannot be directly attributed to arsenic exposure under the conditions of this study. Overall, the results highlight the importance of continued monitoring of As in water sources and the need for further studies with larger sample sizes and additional variables to better understand its potential impact on animal health.

## 1. Introduction

Bioaccumulation is defined as the process by which the concentration of a chemical compound increases in an organism relative to its concentration in the environment [1]. Among these substances, heavy metals represent one of the most persistent threats to human, animal, and environmental health, due to their ability to act as cumulative toxins, even at minimal levels [2]. Arsenic (As) is a toxic metalloid of particular concern due to its persistence in the environment and widespread distribution. It can be found in anthropogenic sources such as industrial and agricultural processes and urban waste [3], as well as through natural processes including volcanic activity, erosion, weathering, and volatilization of exudates in soils and sediments. Through rainfall, As reaches soils, rivers, lakes, and seas, making water the main source of exposure [4].

Human exposure to As has increased considerably due to the consumption of contaminated water and food. One of the concerns in the study of inorganic arsenic is that it has been recognized as a carcinogen, which is why the World Health Organization [5] has established a guideline value of 10 µg/L for drinking water [6]. However, this metalloid continues to circulate through aquifers, soils, and food chains, posing risks to both human and animal health [7]. Groundwater contaminated with As promotes the incorporation of this metalloid into the food chain through crop irrigation [8,9]; this is particularly relevant in the Comarca Lagunera region, where more than 80% of the groundwater is used for agriculture and livestock production [10].

The route of As exposure in goats is through the ingestion of contaminated water, fodder, and soil, and milk has been proposed as a potential biomarker of heavy metal exposure in animals [11,12]. Some factors that influence the metabolism and detection of As in the milk of exposed animals include species, diet, health status, and environmental conditions [13,14]. In addition to containing essential minerals, milk can also retain arsenic, so its consumption may represent a potential risk to human health, especially in areas with high environmental exposure [15,16]. In recent years, As concentrations have increased significantly, making its evaluation in livestock of economic importance in the region necessary. Goats were selected as the study species because they play an important role in local dairy production. In addition, goats are known to consume water directly from local sources and may therefore serve as useful bioindicators of environmental exposure to contaminants such as arsenic. In this context, the present study aims to determine the concentrations of As in water and goat milk in the Comarca Lagunera region and to evaluate their possible association with DNA damage in lymphocytes using the comet assay, to better understand exposure levels and potential associated risks.

## 2. Materials and Methods

### 2.1. Study Area

The Comarca Lagunera region is located in northern Mexico, covers an area of 47,980 km^2^, and comprises ten municipalities in the state of Durango (Lerdo, Gómez Palacio, Mapimí, Nazas, Rodeo, Tlahualilo, General Simón Bolívar, San Juan de Guadalupe, San Luis del Cordero, and San Pedro del Gallo) and five in the state of Coahuila (Matamoros, San Pedro, Torreón, Viesca, and Francisco I. Madero) [16].

It is a semi-arid area characterized by water scarcity and a dry climate, with hot temperatures reaching up to 45.3 °C and cold temperatures ranging from 8 °C to 0 °C, and even reaching −7 °C.

The most important waterways in the region are the Nazas River and the Aguanaval River. According to historical records from the Torreón Meteorological Observatory, the period of heaviest rainfall occurs between June and September, with historical monthly averages of 30 mm in June, 42.8 mm in July, 40.9 mm in August, and 51.6 mm in September (Subdelegación de Planeación, 2024). The predominant vegetation in the Comarca Lagunera is xerophytic scrub, as well as xerosol soils [16].

**Figure 1:**
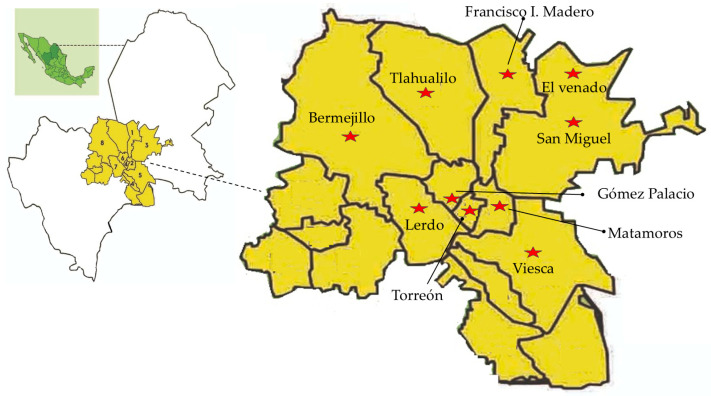
Geographic location of the sampling sites in the municipalities of Gómez Palacio, Lerdo, and Torreón, within the Comarca Lagunera region (Durango and Coahuila, Mexico). Red stars indicate the localities visited during the study (own elaboration).

For this study, 12 locations were visited in the municipalities of Lerdo, Matamoros, San Pedro, Torreón, Mapimí, Gómez Palacio, Tlahualilo, Viesca, and Francisco I. Madero, as shown in Figure 1.

### 2.2. Sampling Design

The evaluation period was from March 8th to April 30th of 2025, during which time the different communities were visited, and samples were taken. A total of 120 female goats (10 animals per herd across 12 locations) were included in the study, considering animals between 2 and 9 years of age, all of which were of the Criollo breed (Table 1). The herds that were sampled are specifically intended for milk production and belong to both intensive and mixed systems, so their diet consists mainly of alfalfa and what they eat while grazing. The animals drink water from containers provided by the producer, which are supplied with water directly from the nearest source. From each animal, blood and milk samples were collected, and one representative water sample was obtained per herd from the main drinking source.

### 2.3. Sample Collection and Processing

Containers previously washed with 10% nitric acid (Sigma-Aldrich, St. Louis, MO, USA) and rinsed with distilled water were used to collect both water and milk samples. Milk samples were collected from each goat in active lactation (~100 mL per animal) directly from the udder and stored at −20 °C. However, for arsenic determination, one representative milk sample per herd was selected for analysis, resulting in a total of 12 milk samples analyzed. This sampling approach limits the ability to evaluate intra-herd variability. Before arsenic determination, milk samples were subjected to acid digestion using nitric acid following standard protocols for trace element analysis. The analysis was performed at the National Laboratory for Water, Soil, Plant, and Atmospheric Analysis of the National Institute of Forestry, Agricultural, and Livestock Research (INIFAP), and samples were analyzed within one month after collection.

For the water samples, one representative sample of 1 L was collected from the drinking source used by each herd and transferred to INIFAP, where it was analyzed through AAnalyst 700 atomic absorption spectrometry. Only one water sample per herd was collected; therefore, statistical comparisons among herds should be interpreted with caution, as this sampling design does not capture temporal or spatial variability. Therefore, water data were treated as descriptive and exploratory, and statistical comparisons among herds should be interpreted with caution.

Blood samples were taken directly from the jugular vein of each goat using sterile Vacutainer tubes with EDTA as an anticoagulant. Approximately 2 mL of blood was obtained from each animal. Samples were kept around 4 °C and transported to the Cell Biology and Ultrastructure Laboratory of the Autonomous University of Coahuila, Faculty of Medicine. Blood samples were analyzed using the alkaline comet assay technique for single cells, following the method originally described by Singh et al. [17,18] with minor modifications. All samples were processed within 2 h after collection.

Quality control procedures included duplicate samples, spiked samples for recovery evaluation, and calibration standards prepared from certified arsenic solutions.

### 2.4. Detection of Arsenic in Milk

Milk samples were subjected to acid digestion following the method described by Cox [19] to mineralize the organic matrix before arsenic determination. Briefly, 5 mL of milk was placed in the digestion vessel and heated at 125 °C for 60 min. A second digestion step was performed by adding nitric acid and heating for 30 min while increasing the temperature to 240 °C until the sample reached near dryness. Subsequently, sulfuric acid (Sigma-Aldrich, St. Louis, MO, USA) was added, and the mixture was heated for 4–6 h until complete mineralization was achieved. The sample was then again brought to near dryness by increasing the temperature to 280 °C, and finally, 1.5 mL of sulfuric acid was added to stabilize the digest. The digested samples were analyzed for arsenic using AAnalyst 700 atomic absorption spectrometer equipped with a hydride generation system (HG-AAS).

### 2.5. Detection of Arsenic in Water

The samples were analyzed using hydride generation atomic absorption spectrometry (HG-AAS) with a palladium-magnesium matrix modifier, following the atomization program recommended in EPA method 206.2.

Table 2 shows the chemometric parameters obtained during the development of the analytical procedure. Detection and quantification limits were calculated according to the Miller and Miller [20] method. The calibration curve showed excellent linearity with a correlation coefficient (R^2^ = 0.9992) within a linear range of 16.3–320 µg/L.

The Limit of Detection (LOD) and limit of quantification (LOQ) were 5.43 µg/L and 16.3 µg/L, respectively. Method precision was 98% with an approximate analytical error 2%. Recovery values obtained from spiked samples were higher than 96%, indicating good accuracy of the analytical method.

### 2.6. Comet Assay

Pre-treated slides with 0.65% normal melting point agarose were prepared, 80 µL of low melting point agarose plus 20 µL of whole blood were added, deposited on the pre-treated 0.65% slides, covered with coverslips, and allowed to solidify at 4 °C for 4 min. The slides were placed in a Coplin jar with lysis solution (2.5 M NaCl, 100 mM EDTA, 10% DMSO, 1% Triton X-100) at 4 °C for 24 h. Electrophoresis was performed at 25 V and 300 mA for 20 min, with a prior resting time of 20 min in electrophoresis solution (300 mM NaOH, 200 mM EDTA). Once electrophoresis was complete, the slides were rinsed with distilled water (laboratory grade) and neutralizing solution (0.4 M Tris-HCl) for 5 min. Finally, they were dehydrated with absolute ethanol and left to dry at room temperature. Once dry, they were stained with GelGreen™ (Biotium, Fremont, CA, USA) 10X dye. The cells were observed under a LABOMED Lx 400 fluorescence microscope (Labomed Inc., Los Angeles, CA, USA) at 40× magnification. Photographs were taken of the different fields where the cells were observed and then submitted to Comet Score 2.0, evaluating 200 cells per individual sample. The mean DNA damage values obtained from the analyzed cells were calculated for each animal and used for the statistical analysis. Therefore, “*n*” represents the number of animals evaluated per herd. The alkaline comet assay was performed following standard procedures described in the literature.

### 2.7. Statistical Analysis

IBM SPSS Statistics version 26 (IBM Corp., Armonk, NY, USA) was used for statistical analysis. Descriptive statistics were performed, calculating the arithmetic mean and standard deviation. Shapiro–Wilk and Kolmogorov–Smirnov tests were performed to assess normality. To determine significant differences between herds, the non-parametric Kruskal–Wallis test was used for data without a normal distribution. Spearman’s rank correlation coefficient was used to evaluate the association between water, milk, and DNA damage parameters. A significance level of *p* < 0.05 was considered for all statistical analyses.

Given the exploratory nature of the study and the limited sample size, the results should be interpreted with caution. In particular, for water arsenic concentrations, statistical comparisons among herds were considered exploratory due to the use of a single sample per herd.

## 3. Results

### 3.1. Arsenic Concentrations in Milk

Arsenic concentrations in goat milk samples were below the limit of quantification (LOQ = 16.3 µg/L) in all analyzed locations (Table 3). Therefore, it was not possible to reliably assess arsenic transfer from drinking water to milk under the conditions of this study.

Although numerical values ranging from 0.0000 to 2.1 µg/L were obtained during the analytical procedure, these values fall below the quantification limit and should be interpreted with caution. The calculated mean concentration was 0.542 µg/L, while the median value was 0.000 µg/L, indicating that more than half of the analyzed samples showed undetectable or extremely low arsenic levels. This pattern reflects that most analytical signals were below the quantification limit and should be interpreted with caution.

The distribution of the data was non-normal (Shapiro–Wilk test, *p* = 0.000) and showed slight positive skewness (skewness = 1.004), indicating a predominance of values below the quantification limit.

The Kruskal–Wallis analysis revealed no statistically significant differences in arsenic levels among the 12 evaluated herds (H = 18.082, df = 11, *p* = 0.080). Although some localities showed slightly higher analytical responses, these values remain below the quantification limit and therefore cannot be interpreted as true differences in arsenic concentration among herds.

Overall, the results indicate that arsenic in goat milk from the evaluated herds was extremely low and below the quantification capability of the analytical method.

### 3.2. Arsenic Concentrations in Water

**Figure 2:**
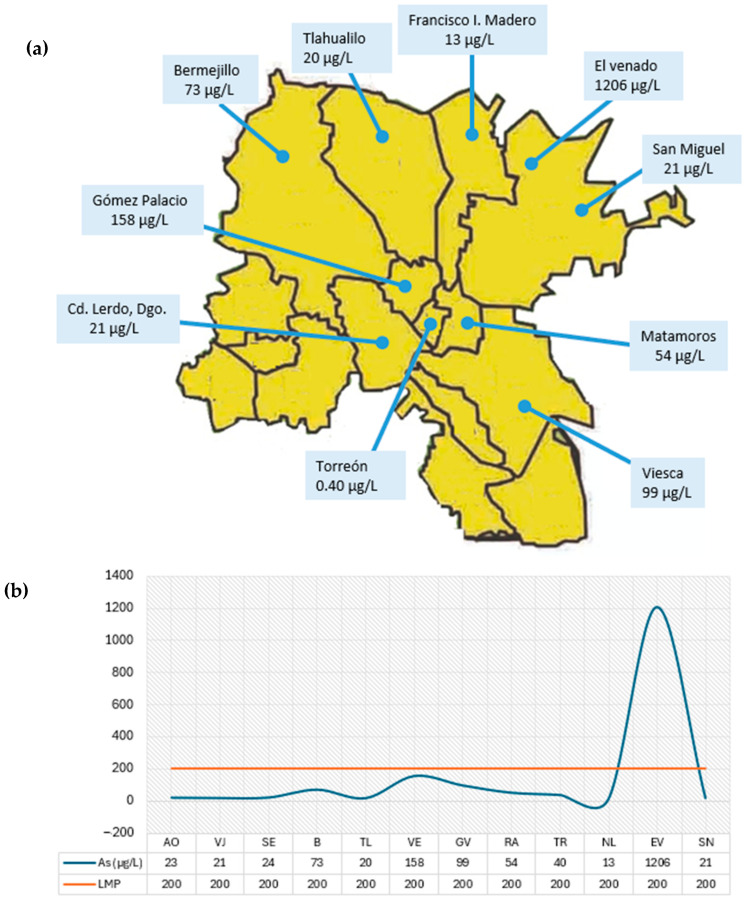
(**a**) Arsenic concentrations in the different locations sampled, (**b**) in relation to the Maximum Permissible Limit (MPL) for animal consumption. AO (Álvaro Obregón), VJ (Villa Juárez), SE (Seis de Enero), B (Bermejillo), TL (Tlahualilo), VE (Venecia), GV (Gabino Vásquez), RA (Redención Agraria), TR (Torreón), NL (Nuevo León), EV (El Venado), SN (San Nicolás) (Own elaboration).

Analysis of drinking water samples revealed wide variability in As concentrations among the communities evaluated (Figure 2a). Notably, the town of El Venado, San Pedro, Coahuila, had the highest arsenic concentration, with a value of 1206 µg/L, significantly exceeding the permissible limit of 200 µg/L established for water intended for animal consumption (Figure 2b).

Other communities had arsenic concentrations close to the permissible limit for animal consumption. However, when considering NOM-127-SSA1-2021, which establishes a limit of 50 µg/L for drinking water, it can be seen that most localities exceed this value, which may represent a potential risk to human and animal health.

Arsenic levels in drinking water averaged 146 µg/L, ranging from 13 µg/L to 1206 µg/L. The standard deviation was 324 µg/L, indicating a high dispersion from the mean. The median was 32 µg/L.

Due to the use of a single water sample per herd, statistical comparisons among locations should be interpreted with caution. Therefore, the analysis of arsenic concentrations in water is presented as descriptive rather than inferential. Although variability in arsenic levels was observed among locations, no robust statistical conclusions can be drawn regarding differences between herds.

The Shapiro–Wilk normality test (W = 0.404, *p* < 0.001) and the Kolmogorov–Smirnov test (D = 0.402, *p* < 0.001) confirmed that the data did not follow a normal distribution (*p* < 0.05).

A Kruskal–Wallis test was performed as an exploratory analysis; however, given the limited replication (one sample per herd), the results should not be considered conclusive (H = 11.000, df = 11, *p* = 0.443).

Higher arsenic concentrations were observed in El Venado, Gómez Palacio, and Viesca. Among these three localities, El Venado exceeded the MPL established by NOM-127-SSA1-2021, while the lowest levels were found in Nuevo León, Francisco I. Madero and Tlahualilo.

### 3.3. Damage to the DNA of Individual Cells (Lymphocytes)

**Figure 3:**
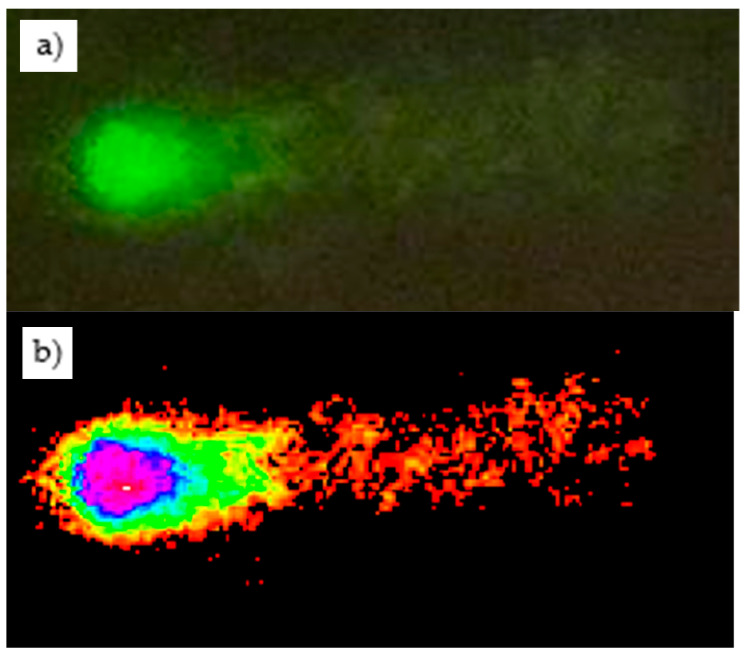
Representative comet assay images obtained from goat lymphocytes. (**a**) Fluorescence micrograph of a lymphocyte stained with GelGreen^TM^ observed at 40× magnification, where the Fluorescent signal corresponds to DNA. (**b**) Image analyzed using CometScore 2.0 software (TriTek Corp., Sumerduck, VA, USA) showing the comet head and tail used to estimate DNA fragmentation; the head represents intact DNA, while the tail indicates fragmented DNA.

DNA damage was observed in some lymphocytes analyzed by the comet assay, as illustrated in the representative images shown in Figure 3.

The percentage of damage i n lymphocytes was measured as % DNA in the head and % DNA in the tail. The results reveal that the average percentage of DNA in the head is 84.79%, with the lowest possible value of 70.99% and the highest of 91.61%, while the average DNA fragmentation on the tail is 15.21%, with values ranging from 8.38% to 29.01%. In both cases, the range was 20.63%, and the standard deviation of ±3.40 showed moderate variation among the samples analyzed.

These findings indicate that, in most of the reviewed cells, DNA remains condensed within the nucleus. However, we observed percentages of tail DNA in some cells that were close to 30% (Table 4).

The number of animals analyzed per locality varied due to sample quality or processing constraints.

Data were analyzed with the Shapiro–Wilk and Kolmogorov–Smirnov tests to verify their normality compared to a theoretical normal distribution. In this case, the two tests indicated values of *p* < 0.05, which shows us that the variable % DNA in tail and head do not follow a normal distribution.

The Kruskal–Wallis test indicated that there are statistically significant differences between at least two herds in the percentages of fragmentation of total DNA (H = 51,851, df = 11, *p* < 0.001). Tlahualilo and Torreón had the highest DNA integrity, while San Nicolás and Seis de Enero showed greater fragmentation.

**Figure 4:**
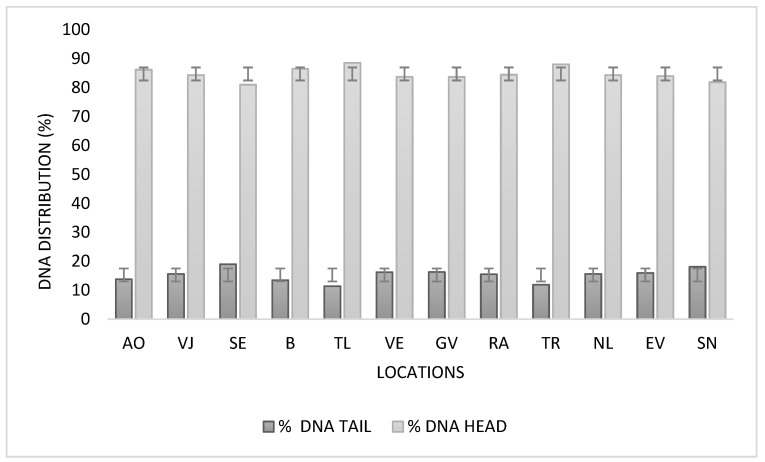
Percentage of DNA damage in lymphocytes evaluated by the comet assay for each sampled batch. Dark bars represent the percentage of DNA in the tail (DNA fragmentation), and light bars represent the percentage of DNA in the head (DNA integrity). Error bars represent standard deviation. AO (Álvaro Obregón), VJ (Villa Juárez), SE (Seis de Enero), B (Bermejillo), TL (Tlahualilo), VE (Venecia), GV (Gabino Vásquez), RA (Redención Agraria), TR (Torreón), NL (Nuevo León), EV (El Venado), SN (San Nicolás).

The herd evaluated in the locality of Seis de Enero was the one that presented the highest percentage of fragmentation in the DNA (Figure 4).

The association between the average of arsenic concentrations in milk and the percentage of DNA tail in lymphocytes was evaluated by a non-parametric Spearman correlation. A weak positive correlation was observed (r = 0.333); however, this correlation was not statistically significant (*p* = 0.290), the small sample size (*n* = 12), and the predominance of values below the LOQ limits the reliability of this analysis (Table 5).

**Figure 5:**
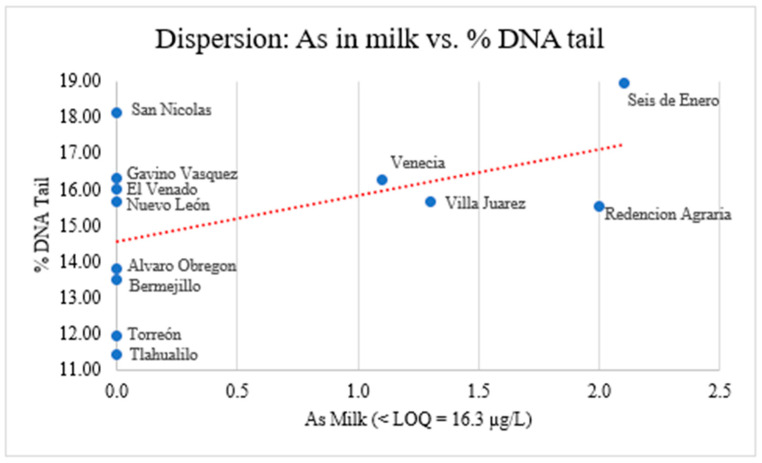
Relationship between DNA damage and arsenic concentration in milk, showing no statistically significant association and values close to the limit of quantification (LOQ) in most sampling locations.

A possible reason for the absence of a statistically significant association might be the small sample size. However, arsenic concentration in milk was below the limit of quantification (LOQ) in most sampling locations, which limits the ability to establish a reliable quantitative relationship. Although slightly higher analytical signals were observed in some locations (Seis de Enero, Villa Juárez, and Redención Agraria), these values should be interpreted with caution, as they remain close to the analytical limits of the method. Overall, no statistically significant association was observed between arsenic levels in milk and DNA damage (Figure 5).

Given the complexity of arsenic metabolism in animals and the influence of multiple factors on cell damage, it is suggested that m ilk could function as a bioindicator of environmental exposure but not necessarily as a direct reflection of the genotoxic effect.

Spearman correlation analysis showed a correlation coefficient of r = 0.207 with a *p* = 0.519 (*n* = 12), indicating no statistically significant association, and suggesting that arsenic levels in water were not related to DNA fragmentation under the conditions of this study (Table 6).

**Figure 6:**
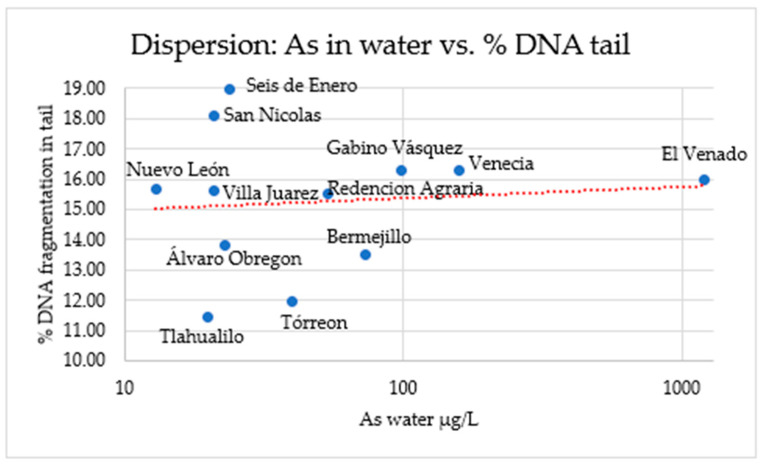
Relationship between As in water and % DNA fragmentation in the tail. The red dashed line indicates the trendline of the correlation.

There is a slight positive trend between As in water and genetic damage. However, although the dispersion among the data is high, some locations, such as El Venado and Venecia, show high As values and slightly high % DNA tail, which could indicate risk areas that warrant monitoring (Figure 6).

## 4. Discussion

This study evaluated arsenic concentrations in drinking water and goat milk, as well as their possible association with DNA damage in lymphocytes of goats from the Comarca Lagunera, Mexico.

Some studies conducted in the Comarca Lagunera have reported variations in As concentration among different areas. The highest levels have been found in communities such as Benito Juárez and El Venado, while other research indicates that Gómez Palacio and Viesca are among the most contaminated municipalities, in contrast to Torreón and Mapimí, which show the lowest concentration [21,22]. Our results suggest that Gómez Palacio and Viesca showed relatively higher arsenic levels after the community of El Venado, which exceeded the MPL established by NOM-127-SSA1-2021, and that the lowest levels were found in communities in Nuevo León, in Francisco I Madero and Tlahualilo.

The absence or very low concentrations of arsenic detected in goat milk, with most samples below the limit of quantification (LOQ), limit the ability to evaluate arsenic transfer into milk. Although the mammary gland in small ruminants may act as a partial barrier to the transfer of certain heavy metals, the predominance of values below the LOQ introduces uncertainty and prevents definitive conclusions regarding arsenic excretion through milk under the conditions of this study.

Regarding the evaluation of genetic damage, the results indicate mild to moderate DNA fragmentation, which could be associated with chronic exposure to environmental stressors. However, a direct causal relationship between arsenic exposure and the observed DNA damage cannot be established under the conditions of this study.

The results of the Kruskal–Wallis test indicate that there are statistically significant differences between at least two herds in the percentages of fragmentation of top DNA (H = 51.851, df = 11, *p* < 0.001). The localities of Tlahualilo and Torreón have the highest ranges, indicating higher proportions of intact DNA, in contrast to San Nicolás and Seis de Enero, which have the lowest and show a greater fragmentation. The herd evaluated in the locality of Seis de Enero was the one that presented the highest percentage of fragmentation in the DNA, which suggests the influence of other environmental or synergistic agents such as other heavy metals (e.g., lead or cadmium), nutritional deficiencies, or oxidative stress conditions that may increase cellular susceptibility to DNA damage.

Although the Kruskal–Wallis test indicated statistically significant differences among herds, pairwise comparisons did not reveal consistent differences between all locations. This may be explained by the high variability within herds and the limited sample size, which can reduce the statistical power despite the presence of extreme values in some localities.

This is consistent with previous studies that mention that arsenic can induce oxidative stress and DNA damage; the absence of significant correlations in this study limits the ability to support this association under the present conditions. This variability reflects individual differences in the biological response to arsenic, possibly due to physiological components, age, diet, exposure time, or detoxification capacity [23,24]. Another study mentions that the genotoxic effect of arsenic depends not only on the dose but also on the time of exposure, species, age, sex, genetics, and nutritional aspects, as well as the interaction with other compounds present in the environment [25]. This reinforces the need to consider multiple variables when assessing the impact of arsenic on animal health. Therefore, the implementation of longitudinal studies, the inclusion of other biomarkers (such as oxidative stress or apoptosis indicators), and joint exposure analysis with other contaminants are recommended to better understand the dynamics of observed cell damage.

Some studies have shown that the genotoxicity of arsenic depends on both concentration and exposure time, and that low or moderate levels may not result in genetic damage detected by some techniques [26,27]. Other contaminants, including lead, as well as other factors such as oxidative stress or nutritional status, could be modifying DNA damage, making it difficult to attribute specifically to As exposure. In addition, evidence shows that co-exposures and antioxidant conditions play a significant role in genotoxic damage [28]. It is also important to consider that although the comet assay is a sensitive tool for detecting DNA damage, it may not capture subclinical or cumulative alterations that require complementary techniques for detection [29].

The absence of statistically significant correlations between arsenic concentrations in water or milk and the comet assay parameters indicates that, within the limitations of this dataset, the study remains primarily descriptive rather than conclusive. Although arsenic exposure has been widely associated with genotoxic effects, the DNA damage observed in goat lymphocytes cannot be directly attributed to arsenic exposure under the conditions of this study. This suggests that the DNA damage observed in goat lymphocytes may not be exclusively attributable to arsenic exposure. Other environmental factors, including exposure to additional contaminants, oxidative stress, nutritional status, or management conditions, may also contribute to the observed variability in DNA damage. Therefore, while arsenic contamination remains an important environmental concern in the Comarca Lagunera region, the results should be interpreted cautiously, considering the potential influence of multiple interacting factors.

An interesting observation in the present study is the contrast between the localities of El Venado and Villa Juárez. El Venado presented the highest arsenic concentrations in drinking water, yet lower levels of DNA damage were observed compared to Villa Juárez, where arsenic concentrations were relatively lower. This apparent discrepancy may be related to factors such as differences in arsenic bioavailability or the presence of other elements in groundwater that may exert antagonistic effects, as well as other environmental pollutants, oxidative stress conditions, and nutritional status. Another limitation of this study is that only one water sample was collected per herd, which may not fully represent temporal variations in arsenic exposure. This limitation restricts the ability to perform robust statistical comparisons among herds and reduces the representativeness of arsenic exposure, as temporal and spatial variability in water sources was not captured. In addition, other environmental contaminants were not analyzed, which limits the ability to attribute the observed DNA damage specifically to arsenic.

Furthermore, the study did not include a non-exposed control group from a region without arsenic contamination. Future studies incorporating reference populations would allow a more accurate interpretation of baseline DNA damage levels.

Another limitation of the study is the uneven age distribution of the sampled animals, as older goats were underrepresented in the dataset. Age may influence physiological responses to environmental contaminants and DNA damage.

## 5. Conclusions

This study identified As in drinking water, with some locations exceeding permissible limits for animal and human consumption. The highest concentration was detected in the community of El Venado, San Pedro, Coahuila. However, arsenic concentrations in goat milk were generally low, with most values below the limit of quantification, which limits the ability to assess arsenic transfer into milk, although very low levels were detected in some samples.

Although increased DNA fragmentation was observed in some localities, the correlations between arsenic exposure and DNA damage were weak and not statistically significant. Therefore, the observed DNA damage cannot be directly attributed to arsenic exposure and may be influenced by other environmental or biological factors. Further studies with larger sample sizes and the inclusion of additional environmental contaminants are required to better understand the relationship between arsenic exposure and genotoxic effects in goats.

## Figures and Tables

**Table 1:** The ages of each of the goats sampled in different locations.

Locations	Age of Animals									Average/Age
	2 Years	3 Years	4 Years	5 Years	6 Years	7 Years	8 Years	9 Years	Total	
Seis de Enero	2	5	2	1	0	0	0	0	10	3.2
Nuevo León	0	0	3	2	1	1	2	1	10	6
Venecia	0	0	1	2	6	1	0	0	10	5.7
Álvaro Obregón	1	3	2	3	1	0	0	0	10	4
Torreón	1	1	4	2	1	1	0	0	10	4.4
Gabino Vásquez	0	0	0	0	5	5	0	0	10	6.5
Villa Juárez	1	5	1	1	2	0	0	0	10	3.8
Redención Agraria	0	3	1	4	2	0	0	0	10	4.5
Tlahualilo	0	0	0	3	5	2	0	0	10	5.9
San Nicolás	0	0	0	1	9	0	0	0	10	5.9
Bermejillo	0	0	1	5	4	0	0	0	10	5.3
El Venado	0	0	0	1	4	5	0	0	10	6.4
**Total**	5	17	15	25	40	15	2	1	120	

**Table 2:** Chemometric parameters of the methodologies used.

Element	Arsenic
Unit	µg/L
Correlation	0.9992
Curve Equation	Y = 0.0248 + 0.0026X
LOD	5.43
LOQ	16.3
Linear Range	16.3–320
Precision	98
% Error	2
% Recovery	>96.0

LOD: Optimal limit of detection, LOQ: Optimal limit of quantification.

**Table 3:** Arsenic concentrations in goat milk samples.

Location	As µg/L	SD	Mean
Álvaro Obregón	<LOQ	-	-
Villa Juárez	<LOQ	-	-
Seis de Enero	<LOQ	-	-
Bermejillo	<LOQ	-	-
Tlahualilo	<LOQ	-	-
Venecia	<LOQ	-	-
Gabino Vásquez	<LOQ	-	-
Redención Agraria	<LOQ	-	-
Torreón	<LOQ	-	-
Nuevo León	<LOQ	-	-
El Venado	<LOQ	-	-
San Nicolás	<LOQ	-	-

Note: LOQ = 16.3 µg/L.

**Table 4:** Percentage of damage in individual cells (lymphocytes).

Locality	*n*	% DNA Head	SD (Head)	% DNA Tail	SD (Tail)	Olive Tail Moment	SD (Olive)
Álvaro Obregón	10	86.19	1.9	13.81	1.9	1.220	0.300
Villa Juárez	10	84.37	2.7	15.63	2.7	1.093	0.131
Seis de Enero	9	81.05	5.0	18.95	5.0	1.332	0.349
Bermejillo	10	86.51	1.9	13.49	1.9	1.377	0.195
Tlahualilo	10	88.57	1.6	11.43	1.6	1.211	0.238
Venecia	9	83.73	2.8	16.27	2.8	1.959	0.525
Gabino Vásquez	10	83.69	2.7	16.31	2.7	1.812	0.761
Redención Agraria	9	84.50	2.8	15.50	2.8	0.976	0.187
Torreón	10	88.06	2.6	11.94	2.6	1.094	0.130
Nuevo León	8	84.34	1.0	15.66	1.0	1.079	0.147
El Venado	10	84.02	1.6	15.98	1.6	1.777	0.230
San Nicolás	10	81.89	3.8	18.11	3.8	1.554	0.588

**Table 5:** Spearman correlation coefficient between the As in milk and the percentage of DNA in the tail.

**Correlations**				
			As_milk	DNA_Tail
Spearman’s Rho	As_milk	Coefficient of correlation	1.000	0.333
		Significance (two-tailed)	—	0.290
		N	12	12
	DNA_Tail	Coefficient of correlation	0.333	1.000
		Significance (two-tailed)	0.290	—
		N	12	12

**Table 6:** Spearman correlation coefficient between the As in water and the percentage of DNA fragmentation in the tail.

Correlations				
			As_water	DNA_Tail
Spearman’s Rho	As_water	Coefficient of correlation	1.000	0.207
		Significance (two-tailed)	-	0.519
		N	12	12
	DNA_Tail	Coefficient of correlation	0.207	1.000
		Significance (two-tailed)	0.519	-
		N	12	12

## Data Availability

All data generated and analyzed during this study are available from the corresponding author upon reasonable request.

## Abbreviations

The following abbreviations are used in this manuscript:

AAS	Atomic Absorption Spectrometry
AO	Alvaro Obregon
As	Arsenic
B	Bermejillo
C	Colorimetry
DMSO	Dimethyl Sulfoxide
DNA	Deoxyribonucleic Acid
EDTA	Ethylenediaminetetraacetic acid
EV	El Venado
GV	Gabino Vasquez
HCl	Hydrochloric Acid
HG	Hydride Generation System
INIFAP	National Institute of Forestry, Agricultural and Livestock Research
LOD	Optimal Limit of Detection
LOQ	Optimal Limit of Quantification
NaCl	Sodium Chloride
NaOH	Sodium Hydroxide
NL	Nuevo León
RA	Redención Agraria
SE	Seis de Enero
SN	San Nicolás
TL	Tlahualilo
TR	Torreón
VE	Venecia
VJ	Villa Juárez
